# Networked collective intelligence improves dissemination of scientific information regarding smoking risks

**DOI:** 10.1371/journal.pone.0227813

**Published:** 2020-02-06

**Authors:** Douglas Guilbeault, Damon Centola

**Affiliations:** 1 The Annenberg School for Communication, University of Pennsylvania, Philadelphia, Pennsylvania, United States of America; 2 School of Engineering, University of Pennsylvania, Philadelphia, Pennsylvania, United States of America; Ca' Foscari University of Venice, ITALY

## Abstract

Despite substantial investments in public health campaigns, misunderstanding of health-related scientific information is pervasive. This is especially true in the case of tobacco use, where smokers have been found to systematically misperceive scientific information about the negative health effects of smoking, in some cases leading smokers to increase their pro-smoking bias. Here, we extend recent work on ‘networked collective intelligence’ by testing the hypothesis that allowing smokers and nonsmokers to collaboratively evaluate anti-smoking advertisements in online social networks can improve their ability to accurately assess the negative health effects of tobacco use. Using Amazon’s Mechanical Turk, we conducted an online experiment where smokers and nonsmokers (N = 1600) were exposed to anti-smoking advertisements and asked to estimate the negative health effects of tobacco use, either on their own or in the presence of peer influence in a social network. Contrary to popular predictions, we find that both smokers and nonsmokers were surprisingly inaccurate at interpreting anti-smoking messages, and their errors persisted if they continued to interpret these messages on their own. However, smokers and nonsmokers significantly improved in their ability to accurately interpret anti-smoking messages by sharing their opinions in structured online social networks. Specifically, subjects in social networks reduced the error of their risk estimates by over 10 times more than subjects who revised solely based on individual reflection (*p* < 0.001, 10 experimental trials in total). These results suggest that social media networks may be used to activate social learning that improves the public’s ability to accurately interpret vital public health information.

## Introduction

Tobacco use continues to be one of the leading preventable causes of death worldwide, and yet public misunderstanding about the adverse health effects of smoking is widespread [1–4]. Both smokers and nonsmokers have been shown to be surprisingly inaccurate in their assessment of the negative health impact of tobacco use [3, 5–8]. A large number of studies attempt to correct misinterpretations of tobacco use through carefully designed anti-smoking campaigns, based on psychological theories of how messages influence the attitudes, beliefs, and behaviors of individuals [9–14]. While a breadth of studies report positive effects of anti-smoking campaigns across a range of countries [5, 14–18], a number of studies also report inconsistent [11, 18–21] or counterproductive outcomes [22–25], where exposure to anti-smoking messages has been shown to unexpectedly strengthen smokers’ pro-smoking bias, and, as a result, increase the prevalence of smoking behavior. A major problem for public health campaigns is that smokers exhibit biased reasoning [6, 26–32] in response to anti-smoking messages, leading to systematic misinterpretations of public health information.

Several studies have attempted to prevent biased responses to health campaigns by using psychologically informed techniques of message design [3, 9, 12, 14, 21]. A key limitation of this approach is that individual-level psychological theories are often unable to account for social network effects on the spread and interpretation of messaging campaigns [33–35]. The public interpretation of health campaigns, and of anti-smoking campaigns in particular, has been shown to be highly mediated by discussion in peer networks, both online [4, 36, 37] and offline [38]. Especially concerning is the finding that smokers largely discuss tobacco-related information in homogenous social networks (also called ‘echo chambers’) [39, 40], consisting mainly of fellow smokers who reinforce each other’s pro-smoking biases [4, 41–44]. Some studies suggest that these misinterpretations can be eliminated by social networks that facilitate communication between both smokers and nonsmokers [20, 38, 40, 45, 46]. However, a number of studies have shown that network interactions between smokers and nonsmokers can further amplify biased reasoning, due to intergroup prejudice and defensive responses to smoking-related stigma [47–50]. The identification of these unexpected and counterproductive network effects has inspired a burgeoning research agenda focusing on how social networks–and especially online social networks–can be structured to minimize bias in the interpretation of anti-smoking messages, and of public health information more generally [45, 51–56].

Recently, a growing body of research in collective intelligence has shown how structured social media networks can be used to improve people’s capacity to accurately interpret scientific information [57], even when this information is associated with significant individual-level biases (e.g. motivated reasoning due to political partisanship) [40]. Importantly, this research illustrates how structured, cross-party communication networks can significantly enhance social learning, leading to the elimination of partisan biases on contentious political topics such as climate change [40]. Here, we build on this prior work to test the hypothesis that enabling smokers and nonsmokers to exchange information in structured social networks while evaluating anti-smoking messages can significantly improve their ability to accurately assess the health risks associated with tobacco use. In particular, we test this hypothesis using a novel online social media platform that allowed us to experimentally control whether subjects interpreted anti-smoking messages on their own or with exposure to peer influence in structured social networks.

## Materials and methods

This research was approved by the Institutional Review Board at the University of Pennsylvania, where the study was conducted. All subjects provided their smoking habits and informed consent during registration. Subjects reported their smoking habits by selecting one of these options in response to the following question, “How often do you smoke cigarettes?”: “Regularly”, “Casually”, “I quit”, “I have never smoked”. In following with standard self-report measures of smoking behavior [3, 12, 14, 22], we considered anyone who reported smoking either “regularly” or “casually” as a smoker, and only people who reported “I have never smoked” were treated as nonsmokers [3, 14, 22]. Anyone who selected “I quit” was not invited to the study.

**Fig 1:**
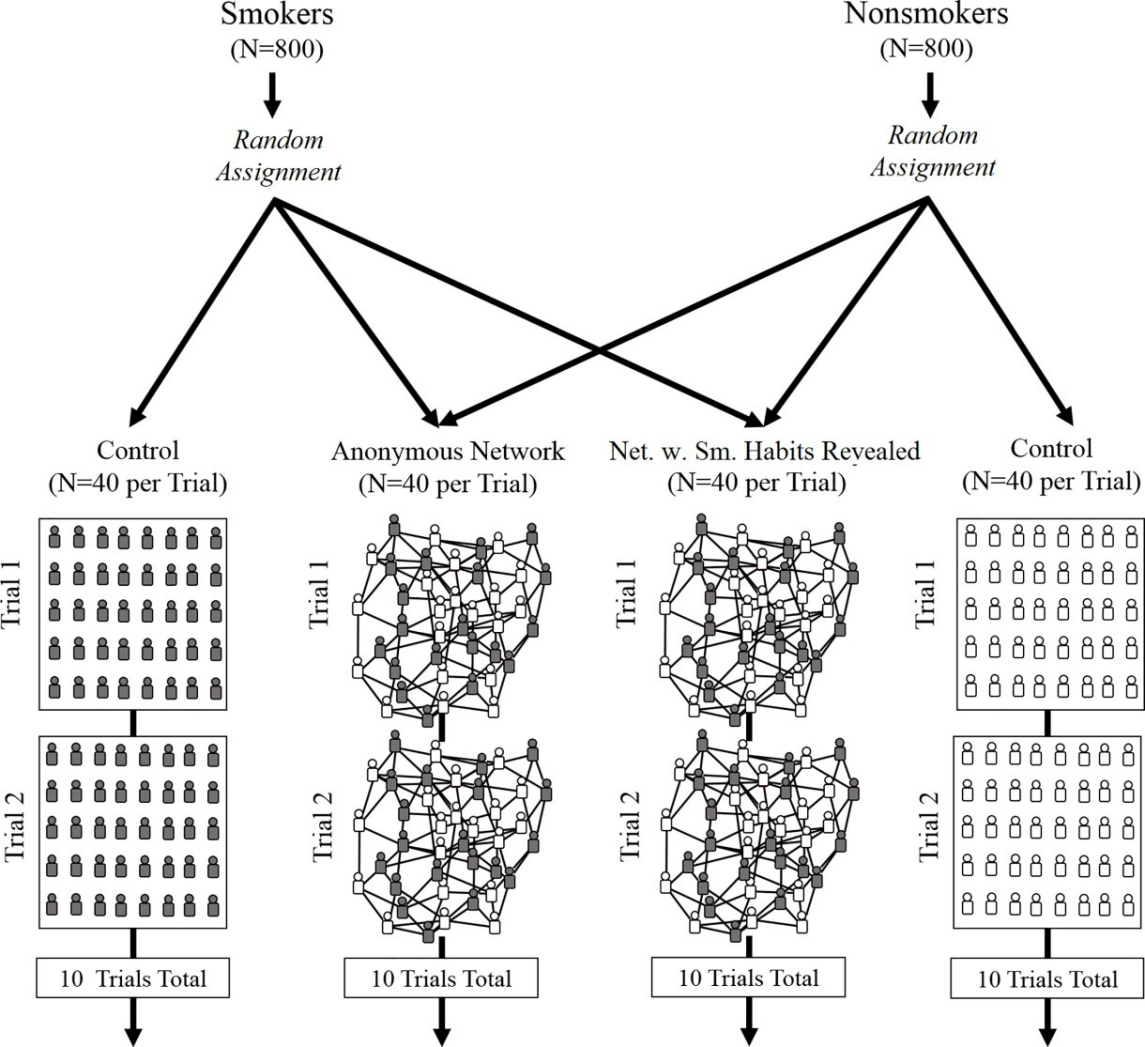
A schematic representation of the experimental design. 800 unique smokers and 800 unique nonsmokers were randomly assigned to one of three conditions: (1) a control condition where they interpreted the anti-smoking messages on their own; (2) an anonymous social network consisting of equal numbers of smokers and nonsmokers, where subjects exchanged views with peers for whom they were given no identifying information; and (3) a social network consisting of equal numbers of smokers and nonsmokers, where subjects exchanged views with peers while being aware of each other’s smoking status as either ‘smoker’ or ‘nonsmoker’. Every condition, in every trial, consisted of 40 unique subjects. Net. w. Sm. Habits Revealed, network with smoking habits of peers revealed.

Upon arriving at the study, participants viewed instructions on how to play an online estimation game (*S1 Appendix*). When a sufficient number of subjects arrived, subjects were randomized to a condition and the trial would begin. We recruited 1,600 unique participants from Amazon’s Mechanical Turk for this study. We did not find any significant differences among smokers and nonsmokers in their demographic traits (*S1 Appendix*). In each trial, subjects were randomized into one of three experimental conditions (Fig 1): (1) a control group of participants who were not placed into a social network (unique control groups of 40 persons were created for both smokers and nonsmokers, requiring 80 subjects per trial); (2) a networked group of 40 persons (20 smokers and 20 nonsmokers) embedded into an integrated (smoker and nonsmoker) anonymous social network, in which participants could observe the opinions of their network contacts; or (3) a networked group of smokers and nonsmokers identical to Condition 2 (i.e., a networked group of 20 smokers and 20 nonsmokers), except that participants could observe their contacts’ smoking status (i.e., “smoker” or “nonsmoker”). The only difference between Condition 2 and Condition 3 is that in Condition 3 participants were shown the usernames of their four network neighbors along with the smoking status of each contact. Usernames were masked and standardized for all players to prevent coordination outside of the experiment (*S1 Appendix*). Fig 1 shows a schematic representation of this experimental design.

We conducted 10 independent trials of this design, requiring 1,600 persons in total. This sample size was chosen based on power testing [58] for the Wilcoxon rank sum test, while incorporating the expected effect size estimated from prior work on social learning in peer networks of the same size and structure as deployed in our experiment [40]. In the control condition, subjects were isolated and not embedded in social networks. In the network conditions, subjects were randomly assigned to a single location in a decentralized, random social network with a uniform degree distribution, in which every subject had four network neighbors (*S1 Appendix*). Using a uniform degree distribution ensured that no one had greater power over the communication dynamics of the network. Maintaining the same topology across network conditions (i.e. Conditions 2 and 3) enabled us to isolate the effects of revealing individuals’ smoking status on the dynamics of intergroup social learning. Every condition contained an equal number of smokers and nonsmokers.

**Fig 2:**
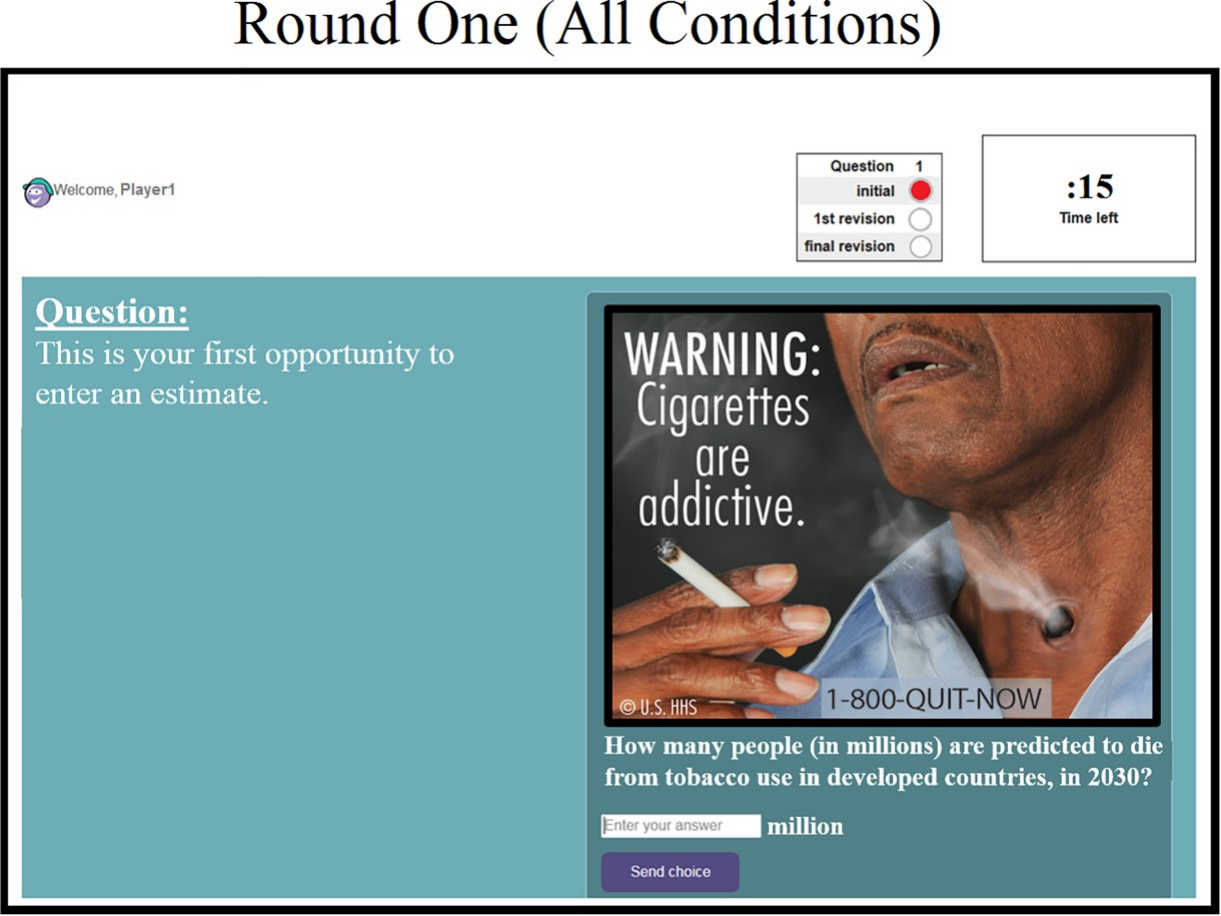
Anti-smoking warning label used as a stimulus in the experiment. This warning label was produced by the U.S. Department of Health & Human Services (© U.S. HHS) in 2011. The question that we used to elicit subjects’ judgements concerning the health risks of tobacco use was taken from the World Health Organization’s report on the global tobacco epidemic, 2015.

In all trials, subjects were exposed to an anti-smoking advertisement from the U.S. Department of Health & Human Services (DHHS) that was released in 2011 [59]. Underneath the advertisement on the experimental web interface, subjects were asked to estimate the health risks of tobacco use by answering the following question–“How many people (in millions) are predicted to die from tobacco use in developed countries, in 2030?”–a question taken from the World Health Organization’s report on the global tobacco epidemic [1] (Fig 2). Subjects in all conditions were awarded monetary prizes based on the accuracy of their final estimates. Accuracy was determined by how close their final answer was to the correct answer provided by the World Health Organization (the correct answer is 30 million people) [1]. Because participants in every experimental condition were equally incentivized to focus on accuracy, economic motivation for accuracy cannot account for any differences in performance across experimental conditions. (Robustness trials were run with additional anti-smoking advertisements. All results were consistent with our main findings; *S1 Appendix*).

For each trial, we measured the change in subjects’ accuracy between Round 1 and Round 3 in terms of the numeric distance between their point-estimates and the correct answer. The effects of social learning were determined by comparing the change in point-estimate accuracy from Round 1 and Round 3 between the control condition and network conditions. In every condition, subjects were given three rounds to provide their estimates. During each round, subjects were given 30 seconds to input an estimate, regardless of condition. In Round 1, all subjects in every condition provided an independent estimate. In Round 2 and Round 3, subjects in Condition 1 (i.e., the control condition) were given the opportunity to revise their answers using independent reflection, without any information about their peers’ responses. In Condition 2 (anonymous networks), subjects in Round 1 gave their independent responses, and in Rounds 2 and 3 they were able to revise their responses while being shown the average answers of their network contacts. In Condition 3 (networks with information about contacts), subjects were able to revise their responses while being shown the usernames and smoking behavior of the four peers connected to them in the social network. The experience of subjects in all conditions was identical except for the presence or absence of social information. Thus, any differences in how smokers and nonsmokers estimated the negative health risks of smoking between the control condition and the network conditions can be attributed to their exposure to social information. Additionally, because information about peer estimates was presented in the same way in both network conditions (S1 Fig; *S1 Appendix*), any differences in subjects’ social learning across experimental conditions can be attributed to the effects of revealing the smoking status of their network peers.

In addition, all subjects across all conditions were asked to complete a post-test survey after the estimation task, involving a number of items that measured (1) subjects’ qualitative perceptions of the negative health risks of tobacco use, (2) subjects’ self-reported confidence in their risk assessments, and (3) subjects’ beliefs concerning the capacity of both smokers and nonsmokers to accurately interpret scientific information about smoking (*S1 Appendix*). Specifically, the post-test survey consisted of five questions, which were always presented in the same order, regardless of condition (*S1 Appendix*). Every survey item was selected based on its established use in communications research as an implicit measure of either belief change, or the intention to change behavior (e.g., intention to quit smoking) (*S1 Appendix*) [14]. These survey items complement the behavioral response data (i.e., risk estimation data) collected in our study. This combination of behavioral response data with qualitative self-report data enabled us to identify the effects of networked social learning on participants’ subjective perceptions of smoking risk, as well as changes in their biases toward social contacts based on their contacts’ status as smokers or nonsmokers.

To begin our analysis, we compare the change in estimation accuracy from Round 1 to Round 3 between all conditions. We measured the change in estimation accuracy between Round 1 and Round 3 for the smoker and nonsmoker control groups (Condition 1), the anonymous networks (Condition 2), and the networks with information on peers’ smoking status (Condition 3). Within each group of 40 subjects in the control condition, subjects were independent. However, within each group of 40 in the network conditions, subjects were not independent. To provide a proper comparison across conditions, each group of 40 subjects was treated as a single observation by taking the average response of each group in each Round of the study. By taking the difference in average response in each group, from Round 1 to Round 3, each trial of 160 subjects (40 subjects per condition) is reduced down to four independent observations. Thus, 10 experimental trials yield 10 independent observations for each condition, and 40 independent observations in total. This approach permits a direct comparison between outcomes of the control condition and the network conditions, enabling us to identify the causal effects of peer influence on changes in subjects’ understanding of public health information.

Similarly, to compare subjects’ qualitative survey responses across experimental conditions, we adopted the same analytical approach, in which we calculated the average response for each survey item in each trial, producing 10 trial-level independent observations for each survey item in each experimental condition, and yielding 40 independent observations in total for each survey item. In order to adopt this statistical approach, the possible responses to each question were formatted to an ordinal scale, so that the categorical inputs could be converted to numerically ranked values which could be compared using the Wilcoxon test, and which could then be aggregated at the trial-level to produce independent data points. This analytical approach provides the unique opportunity to gain casual insight into the effects of social network interactions on changes in participants’ subjective perceptions of smoking.

For robustness, we compared our findings using this more conservative trial-level analyses, which permits causal identification, to more statistically powered analytical approaches. As expected, we found consistent results across all analytical techniques (*S1 Appendix*). Additionally, for further insight, we also applied regression techniques to subjects’ responses in both the control and experimental conditions to test for the effects of subjects’ smoking status on their survey responses, while holding the initial and final estimation error of subjects constant across conditions. To apply these regression techniques to the network conditions, we used clustered standard errors grouped at the trial-level to adjust for nonindependence among subjects in the same network. While these correlational analyses are supplementary to our core experimental findings, we include them here because they add greater interpretative depth to our results.

## Results

We begin our analysis with the finding that both smokers and nonsmokers were equally inaccurate at estimating the fatal health risks of tobacco use. In the control condition, after two rounds of revision, there was no significant improvement. Both smokers and nonsmokers failed to increase the accuracy of their risk estimates. By contrast, we found that in both network conditions, two rounds of revision led to a significant reduction in the error of subjects’ risk estimates. Surprisingly, the benefits of social learning in networks were equally important for both smokers and nonsmokers.

### Information exchange among smokers and nonsmokers facilitated social learning in the interpretation of anti-smoking messages

**Fig 3:**
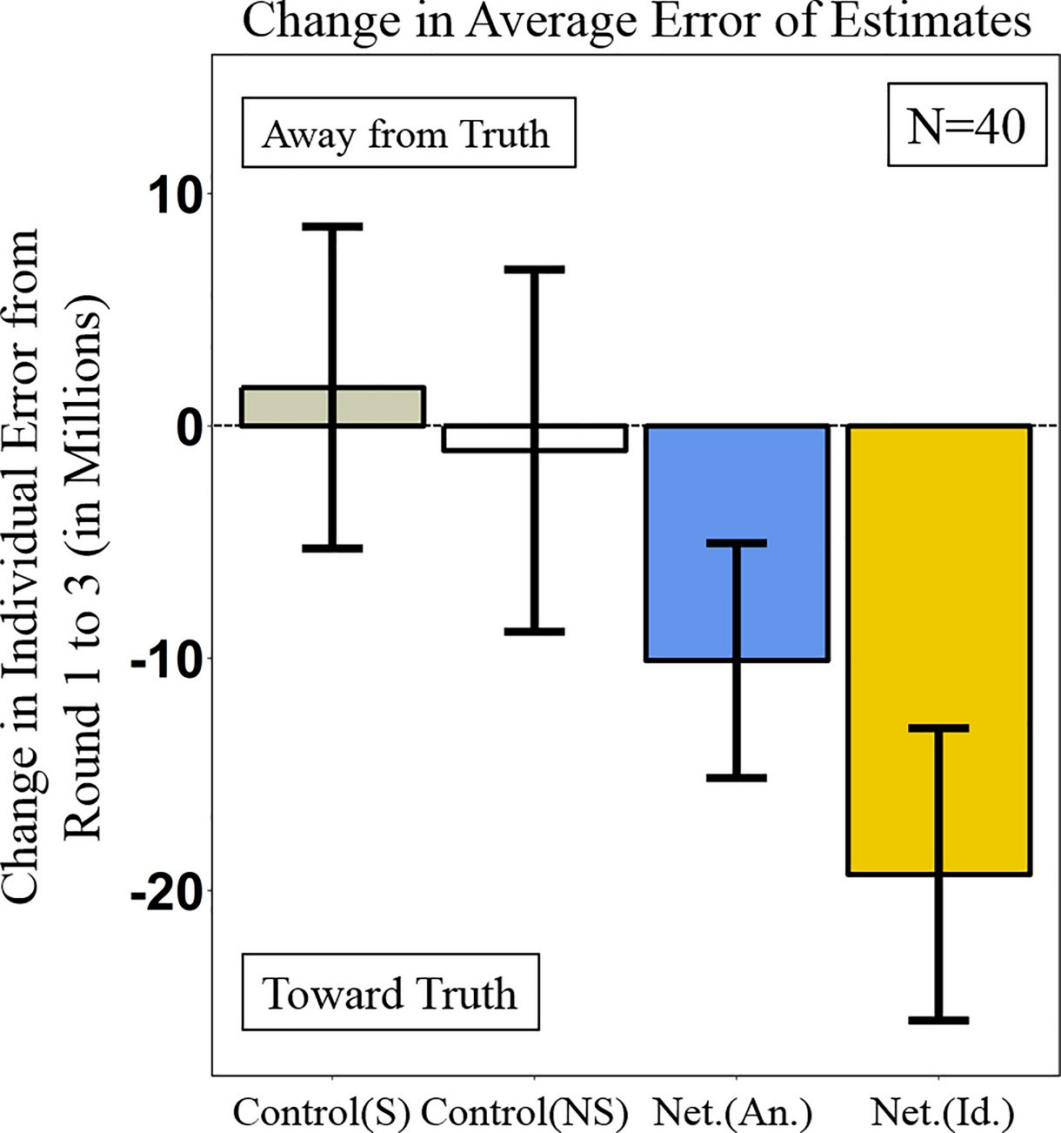
Changes in estimation error across experimental conditions. Bars display the total change in estimation error from Round 1 to Round 3, averaged across all 10 experimental trials, where each trial provides one observation. All conditions are independent. The error bars show 95% confidence intervals. S, smoker; NS, nonsmoker; An., anonymous; Id., with the smoking status of contacts identified.

In Round 1, at baseline, there were no significant differences in the estimates of smokers and nonsmokers in the control condition (*n =* 20, *p =* 0.27, Wilcoxon rank sum test), in the anonymous networks (*n =* 20, *p =* 0.85, Wilcoxon rank sum test), and in the networks with smoking status revealed (*n =* 20, *p =* 0.25, Wilcoxon rank sum test). Importantly, there were no significant differences in the baseline estimation error of smokers across conditions (*n =* 30, *p =* 0.17, Kruskal–Wallis H test), nor in the baseline estimation error of nonsmokers across conditions (*n =* 30, *p =* 0.76, Kruskal–Wallis H test). Fig 3 shows the change in subjects’ estimates from Round 1 to Round 3 in the control and network conditions.

In the control condition, there was no significant change in the accuracy of subjects’ risk estimates, for both smokers (*n =* 10, *p* = 0.69, Wilcoxon signed rank test) and nonsmokers (*n =* 10, *p* = 0.55, Wilcoxon signed rank test), suggesting that their baseline inaccuracies did not improve from individual reflection, and that in some cases the error of these subjects increased. By contrast, in anonymous social networks, the error of subjects’ risk estimates was significantly reduced (by 10.10 million; *n =* 10, *p* < 0.01, Wilcoxon signed rank test). Similarly, in networks with smoking status revealed, there was also a significant reduction in the error of subjects’ risk estimates (by 19.3 million; *n =* 10, *p* < 0.01, Wilcoxon signed rank test).

The total decrease in estimation error among subjects in the anonymous networks was significantly greater than both smokers (by 11.7 million; *n =* 20, *p* = 0.01, Wilcoxon rank sum test) and nonsmokers (by 9 million; *n =* 20, *p* < 0.01, Wilcoxon rank sum test) in the control condition. The total decrease in estimation error among subjects in the networks with smoking habits revealed was over ten times greater than both smokers (by 20.9 million; *n =* 20, *p* < 0.01, Wilcoxon rank sum test) and nonsmokers (by 18.2 million; *n =* 20, *p* < 0.01, Wilcoxon rank sum test) in the control condition. We find that revealing the smoking status of subjects in information-sharing networks led to greater social learning than in the anonymous networks (by 9.2 million; *n =* 20, *p* < 0.01, Wilcoxon rank sum test).

**Fig 4:**
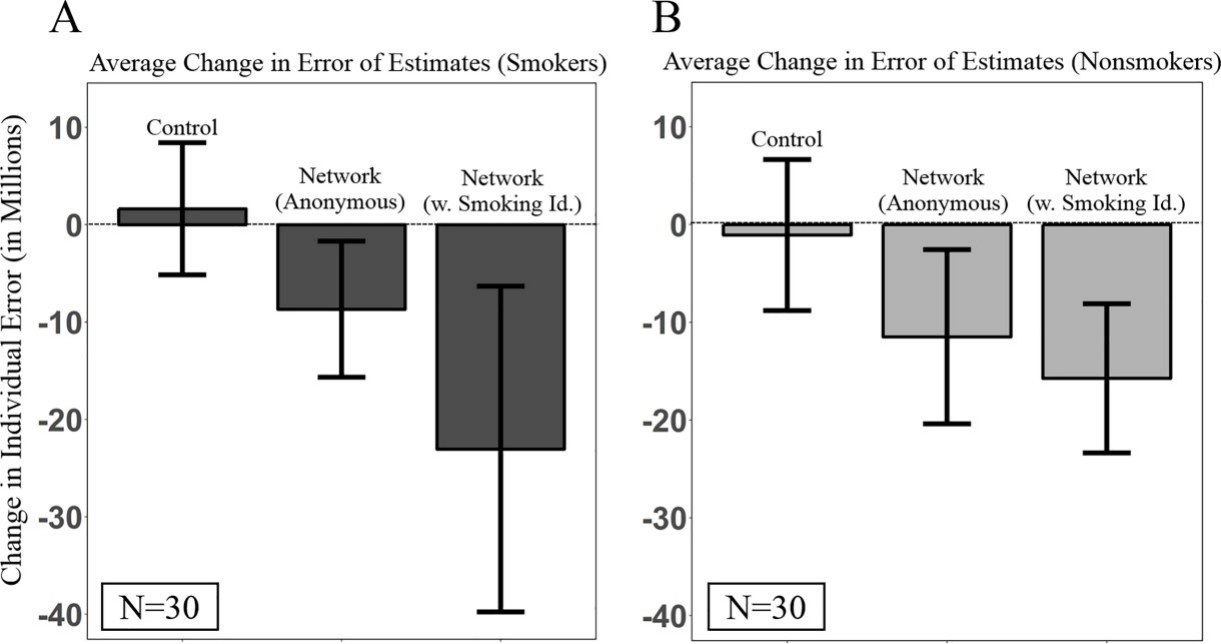
Changes in the average estimation error of individuals across experimental conditions, split by the smoking status of subjects in each condition. (A) The performance of smokers, averaged across all 10 trials, for all conditions. (B) The performance of nonsmokers, averaged across all 10 trials, for all conditions. Network conditions contained 20 smokers and 20 nonsmokers, whereas each control condition contained 40 smokers and 40 nonsmokers. The average estimation accuracy of smokers and nonsmokers in networks was measured separately by computing the average estimation accuracy by each subgroup, within each network, thus producing two group-level observations for each network and 20 in total for each network condition. All conditions are independent. The error bars show 95% confidence intervals. w. Smoking Id., with the smoking status of contacts identified.

Fig 4 shows that the benefits of social learning were equally distributed across both smokers and nonsmokers in the network conditions. Panel A of Fig 4 shows that smokers did not significantly reduce the error of their risk estimations in the control condition (*n* = 10, *p* = 0.69, Wilcoxon signed rank test), while smokers significantly improved their estimation accuracy in the anonymous networks (by 8.67 million; *n* = 10, *p* = 0.02, Wilcoxon signed rank test) and in the networks with the smoking status of social contacts revealed (by 23.03 million; *n* = 10, *p* < 0.01, Wilcoxon signed rank test). Similarly, panel B of Fig 4 shows that nonsmokers did not significantly reduce their estimation error in the control condition (*n* = 10, *p* = 0.55, Wilcoxon signed rank test), while nonsmokers significantly improved their estimation accuracy in the anonymous networks (by 11.46 million; *n* = 10, *p* = 0.02, Wilcoxon signed rank test) and in the networks with the smoking status of peers revealed (by 15.72 million; *n* = 10, *p* < 0.01, Wilcoxon signed rank test).

Remarkably, we find that compared to smokers in the control condition, smokers reduced their estimation error significantly more in the anonymous networks (by 10.31 million; *n =* 20, *p =* 0.05, Wilcoxon rank sum test) and in the networks with smoking habits revealed (by 24.6 million; *n =* 20, *p <* 0.01, Wilcoxon rank sum test), (Fig 4A). Supplementary regression analyses also show that compared to the significant reduction in error from Round 1 to Round 3 exhibited by smokers in both network conditions, in the control condition, independent reflection exacerbated smokers' initial bias, leading to significant increases in error from Round 1 to Round 3 (*S1 Appendix*). Similarly, compared to nonsmokers in the control condition, nonsmokers reduced their estimation error significantly more in the anonymous networks (by 10.39 million; *n =* 20, *p =* 0.03, Wilcoxon rank sum test) and in the networks with the smoking status of peers revealed (by 14.65 million; *n =* 20, *p <* 0.01, Wilcoxon rank sum test), (Fig 4B). There was no significant difference in the total change in estimation error between smokers and nonsmokers in the anonymous networks (*n =* 20, *p =* 0.79, Wilcoxon rank sum test), nor between smokers and nonsmokers in the networks with smoking status revealed (*n =* 20, *p =* 0.52, Wilcoxon rank sum test), suggesting that social learning was equally beneficial to both smokers and nonsmokers.

### Disconnect between confidence and accuracy

To test whether smokers and nonsmokers differed in their perceived confidence, we used regression techniques to control for subjects’ initial and final accuracy in each condition, while using subjects’ smoking status to predict their confidence rating. In our analysis, we clustered standard errors at the trial-level to adjust for nonindependence among subjects in the same networks (*S1 Appendix*). Holding subjects’ initial and final estimation error constant, smokers reported significantly higher confidence levels than nonsmokers in every experimental condition: in the control condition (*n =* 532, *p* < 0.001, CI [0.19, 0.75]), in the anonymous networks (*n =* 334, *p* < 0.001, CI [0.3, 1]), and in the networks with subjects’ smoking status revealed (*n =* 328, *p* < 0.001, CI [0.43, 1.13]).

### Social learning reduced intergroup bias in smokers’ and nonsmokers’ attitudes toward each other

After the estimation task, all subjects in all conditions were asked to rate the extent to which they agreed with the following statement using a 5-point Likert-scale (ranging from Strongly Agree to Strongly Disagree): “Some say that smokers are more likely to *misinterpret* health information about tobacco use than nonsmokers” (*S1 Appendix*). Smokers expressed significantly lower levels of agreement with this statement than nonsmokers in both the control condition (*n =* 20, *p* = 0.01, Wilcoxon rank sum test) and the anonymous networks (*n* = 20, *p* = 0.03, Wilcoxon rank sum test). However, after interacting in social networks with smoking status revealed, there was no significant difference in smokers’ and nonsmokers’ perceptions of smoker’s capacity to interpret health information about tobacco use (*n* = 20, *p* = 0.19, Wilcoxon rank sum test). These results are robust to a range of analytical techniques and statistical tests (*S1 Appendix*).

### Robustness

To test the robustness of our main results, we replicated our experimental design (Fig 1) using two additional anti-smoking advertisements from governmental health organizations (*S1 Appendix*). Across the responses for all questions, we find no significant difference in the initial accuracy between smokers and nonsmokers regardless of the anti-smoking advertisement that was used (*n =* 153, *p* = 0.83, Wilcoxon rank sum test). Consistent with our results, in trials using alternative anti-smoking advertisements, the error of subjects’ risk estimates was significantly reduced in both the anonymous networks (by 8.16 million; *n =* 26, *p* < 0.01, Wilcoxon signed rank test) and the networks with smoking status revealed (by 11.7 million; *n =* 27, *p* < 0.01, Wilcoxon signed rank test). Additionally, across all trials there was no significant difference in the amount of social learning exhibited by smokers and nonsmokers in either the anonymous networks (*n =* 52; *p* = 0.48, Wilcoxon rank sum test) or the networks with smoking status revealed (*n =* 54; *p* = 0.24, Wilcoxon rank sum test). In all replicated trials, both smokers and nonsmokers significantly improved as a result of social network interactions, and improvements were greatest in networks with peers’ smoking status revealed.

## Discussion

Prior research on anti-smoking campaigns has focused largely on how individuals psychologically respond to the design of messages [10, 22], which have yielded inconsistent and even counterproductive effects on tobacco-related beliefs and behaviors [14, 22–25]. To address these concerns, our study identifies how social networks operate to influence the interpretation of anti-smoking advertisements [45]. We find that allowing smokers and nonsmokers to communicate in structured information-sharing networks can generate social learning that significantly improves their ability to accurately interpret the negative health risks of smoking as conveyed by anti-smoking messages. Specifically, we find that subjects in social networks improved in their health-risk estimates by ten times more than subjects in control groups who revised solely based on individual reflection. This magnitude of improvement is likely to be clinically significant, in light of research showing that even minimal improvements in the assessment of smoking risks can have a positive effect on the probability of quitting smoking [60, 61]. Furthermore, in contrast with theories of intergroup bias [38, 47–50, 52], we find that allowing smokers and nonsmokers to exchange views while aware of each other’s smoking status effectively reduces bias both in their evaluation of health risks, and in their beliefs about each other’s capacity to accurately interpret scientific data about the health risks of tobacco use.

A compelling qualitative interpretation of these findings is that individuals who were more accurate in their estimates were also more confident, and therefore more likely to influence their peers [57, 62–64]. To evaluate this possible explanation, before revealing the correct answer, we concluded our study with a qualitative survey asking participants about their confidence in their final answers (*S1 Appendix*). These survey results reveal a surprising disconnect between subjects’ estimation accuracy and their self-reported confidence in their accuracy. Smokers reported substantially higher levels of confidence than nonsmokers across all conditions, while controlling for initial and final accuracy throughout the experiment. These results are consistent with studies suggesting that biased subjects report higher levels of perceived confidence in their prior judgements as a way of resisting the influence of messages aimed at changing their behavior [3, 28–32, 65–68]. Yet, what is most interesting is that despite this disconnect between confidence and accuracy across smokers and nonsmokers, in the network conditions smokers and nonsmokers were nevertheless able to learn from each other and improve their factual understanding of smoking risks. As such, these results suggest that a correlation between confidence and accuracy may not be the correct theoretical construct to account for the remarkable effects of social learning in information-sharing networks [57, 62–64], particularly when individuals may be engaged in motivated reasoning [28–32, 40, 65–68].

Vast amounts of individual variation in media exposure and social network structure, coupled with sparsity in observational data, have imposed major limitations on the capacity for prior studies to examine the causal effects of peer communication on the public interpretation of anti-smoking campaigns. A key strength of this study is that our experimental design and statistical methods afford causal insight into the effects of peer communication on the interpretation of public health campaigns. Particularly important is our ability to identify network conditions in which peer communication can improve the accuracy of public judgements of health campaigns. As such, these findings set the groundwork for future studies that examine the effects of social learning in different network topologies on the public’s capacity to accurately interpret health information.

However, the strengths of our experimental design and results are also accompanied by limitations. To directly measure the effects of peer influence on the interpretation of anti-smoking messages, we restricted communication to numeric estimates regarding smoking risks. While this provided a clear outcome variable when comparing subjects’ assessment of health risks across conditions, it also fails to capture the full range of affective and linguistic modes of communication that play a role in the interpretation of public health campaigns. Nevertheless, it is promising that even when limited to the exchange of numeric estimates, we found that communication led to improvements not only in subjects’ assessment of smoking risks, but also in their willingness to trust the estimates of outgroup members. We expect that our results linking numeric estimates to qualitative judgements regarding smoking risks will help inform future longitudinal studies that examine the effects of social learning on changes in subjects’ health-related behavior–for example, on changes in the rate of smoking cessation among tobacco users.

Altogether, our findings contribute to recent work in the science of science communication on the ability for online social media networks to shape and enhance the public interpretation of vital scientific information. We complement this work by showing show online communication networks can be structured to function as a dynamic filter on people’s judgments that improves their capacity to accurately evaluate the health risks conveyed by public health messages. We anticipate that these findings will provide a useful framework for future research that aims to identify how social media networks can be harnessed to enhance the public understanding of scientific information.

## Supporting information

S1 Appendix Further details on experiment design, subject experience, recruitment, subject demographics, survey design, statistical tests, and additional analyses. (DOCX)

S1 Fig Screenshots of the user interface across all conditions, at Round 2. (TIFF)

S2 Fig Screenshots of the additional anti-smoking messages used in the robustness trials. (TIFF)

S3 Fig Changes in estimation error across experimental conditions for all questions (including robustness questions). (TIFF)

S4 Fig Changes in estimation error across experimental conditions for all questions (including robustness questions), for both smokers and nonsmokers separately. (TIFF)
