# Supplementary material for: Networked collective intelligence improves dissemination of scientific information regarding smoking risks
Source: PLoS One. 2020 Feb 6;15(2):e0227813. doi: 10.1371/journal.pone.0227813 (PMC7004329; doi:10.1371/journal.pone.0227813)
Supplement: S1 Appendix — (DOCX) [file pone.0227813.s001.docx]

Supplementary Information for

**Networked Collective Intelligence Improves Dissemination of Scientific Information Regarding Smoking Risks**

This PDF file includes:

Supplementary Materials and Methods

Supplementary Results

Supplementary Discussion

Supplementary Figures

Supplementary References

**Supplementary Materials and Methods**

**Experiment Design.** In each trial of this study, subjects were randomized into one of three experimental conditions: (1) a control group of participants who were not placed into a social network (unique control groups of 40 persons were created for both smokers and nonsmokers, requiring 80 subjects per trial); (2) a networked group of 40 persons (20 smokers and 20 nonsmokers) embedded into an integrated (smoker and nonsmoker) anonymous social network, in which participants could observe the opinions of their network contacts; or (3) a networked group of smokers and nonsmokers identical to Condition 2 (i.e., a networked group of 20 smokers and 20 nonsmokers), except that participants could observe their contacts’ smoking status (i.e., “smoker” or “nonsmoker”). In each trial, each condition contained 40 individuals, such that each experimental trial contained 160 individuals. We conducted 10 independent trials of this design.

When subjects entered one of the network conditions, they were randomly assigned to one node in the network, and they maintained this position throughout the experiment. The network conditions employed a random network where every node had the same number of connections. We constructed a network with 4 edges per node, and we employed the same network topology across all network conditions to minimize variance. Every network contained an equal number of smokers and nonsmokers. We used random decentralized networks because previous experiments illustrate that this topology is the most reliable for generating social learning in online collective intelligence tasks [1,2].

Subjects were exposed to an anti-smoking message (Fig 2, main text) from the U.S. Department of Health & Human Services (DHHS) that appeared widely as a label on cigarette packaging in 2011 [3]. Underneath the advertisement on the experimental web interface, subjects were asked to estimate the health risks of tobacco use by answering the following question – “How many people (in millions) are predicted to die from tobacco use in developed countries, in 2030?” – a question taken from the World Health Organization’s report on the global tobacco epidemic (WHO, 2015) (Fig 2, main text). Robustness trials were run with additional anti-smoking advertisements. See “Robustness Trials with Additional Anti-smoking Messages”.

**Subject Experience.** To isolate the effect of social influence, the interface was identical across the network and control conditions. When interpreting the anti-smoking advertisement, participants were given two opportunities to revise their answer. The only difference between conditions was that, in the network conditions, subjects were shown peer information while they made their revision, whereas controls were not. In anonymous networks, subjects were shown the average answers of their network neighbors, and then they were permitted to revise their responses. In networks with the smoking status of peers revealed, subjects were not only shown the average answers of their network neighbors, but they were also shown the usernames and smoking status of their network contacts.


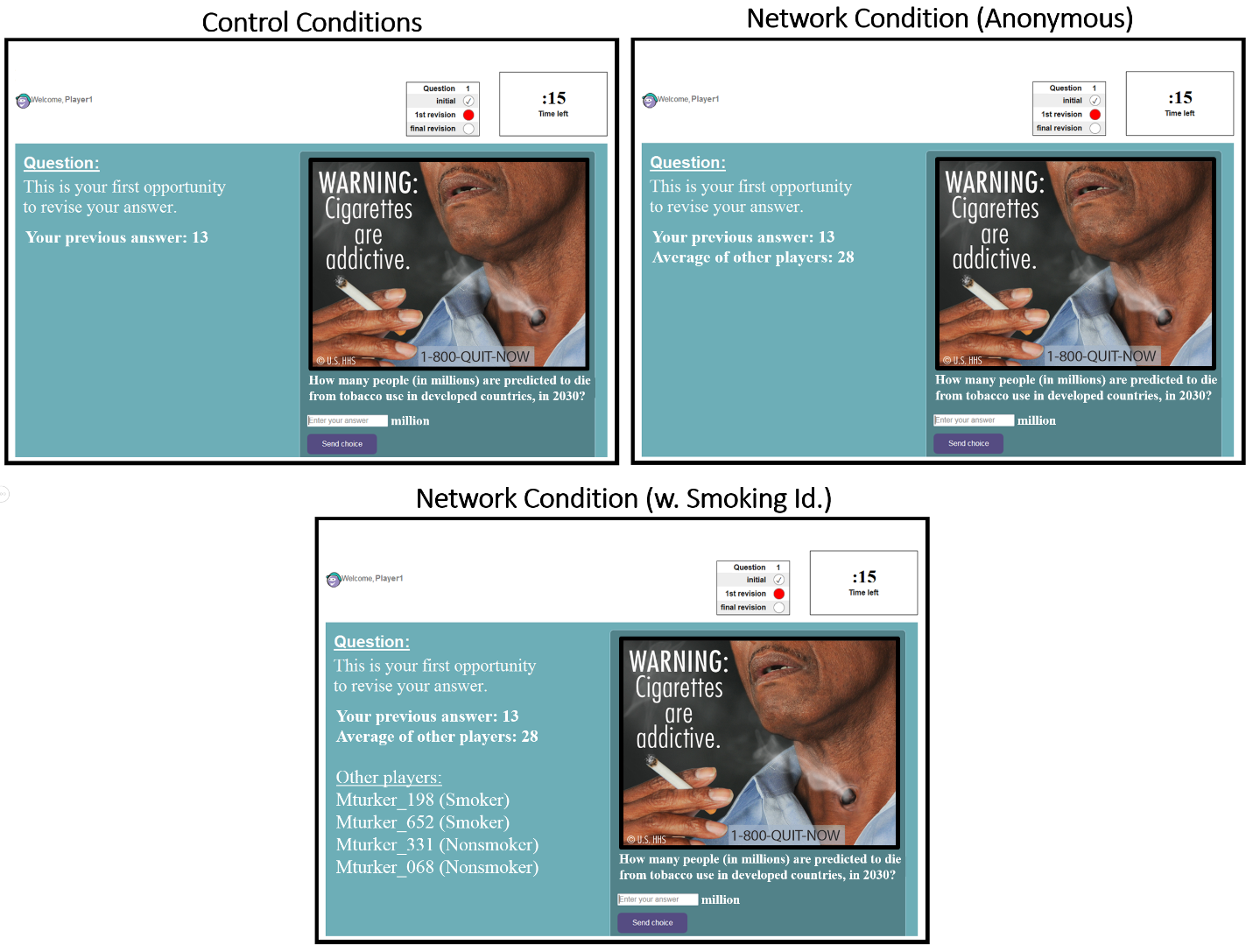


S1 Fig. Screenshots of the user interface across all conditions, at Round 2. w. Smoking Id., with the smoking status of contacts identified. This warning label was produced by the U.S. Department of Health & Human Services (© U.S. HHS) in 2011.

The same standardized usernames were shown to all players so that identity signaling was restricted to smoking status, at the exclusion of variables such as race, age, and gender, which may be signaled by a player’s choice of a username (S1 Fig). The usernames shown were designed to be neutral, so that the choice of username did not reflect additional identity signaling. Also, smoking status did not dictate the order of the names displayed below the group average. This order was determined randomly for each player to avoid order effects (S1 Fig).

Subjects had one minute for each response, and the experiment lasted for five minutes. Subjects were paid based on their error as a percent of the true value. The true answer to the question according to The World Health Organization is 40 million people. Answers which were exactly correct earned the maximum payout ($2.50). Answers which were within 1% of true value received $1.25; within 10%, $1.00; within 15%, $0.75; within 20%, $0.35; within 30%, $0.25; within 90%, $0.15. Answers more than 90% from the true value did not earn payment. This payment schedule was not observable to participants, who were only told "The more accurate your answers, the more you win!" Subjects were not informed about their accuracy and their payments until after the experiment. Designing the interface in this fashion allowed us to isolate the effects of networked information exchange on the capacity for subjects to accurately interpret anti-smoking ads and the health risks they portray.

**Subject Recruitment.** Our participants were recruited over the Amazon Mechnical Turk platform to be players in an “Intelligence Game.” We recruited participants based on their self-identified smoking habits. Subjects reported their smoking habits by selecting one of these options in response to the following question, “How often do you smoke cigarettes?”: “Regularly”, “Casually”, “I quit”, “I have never smoked”. In following with standard self-report measures of smoking behavior, we considered anyone who reported smoking either “regularly” or “causally” as a smoker, and only people who reported “I have never smoked” were treated as nonsmokers. Anyone who selected “I quit” was not invited to the study. Upon registration, subjects provided their smoking habits along with several other demographic questions in random order, including their political partisanship and their education level, such that they were not primed to consider their smoking behavior as an experimental variable of interest in the study.

Upon arriving at the study, participants viewed instructions on how to play the intelligence game, and they waited while other subjects arrived. When a sufficient number of subjects arrived, all subjects were randomized to a condition and the trial would begin. Data were collected over a 1-month period, from January 22^nd^, 2018 to February 2^nd^, 2018. 1,600 participants in total were recruited from the World Wide Web to participate in this study. Exactly half of the participants were self-identified as smokers, and the other half were self-identified as nonsmokers. A quarter of the participants (i.e. 400 subjects) were randomly sorted into each of the main conditions: the smoker control condition, the nonsmoker control condition, the anonymous network condition including both smokers and nonsmokers, and the network condition including both smokers and nonsmokers where the smoking status of peers is revealed.

**Subject Demographics.** After providing their smoking status, subjects were linked to an additional survey that invited them to volunteer more demographic information about themselves. A total of 1106 subjects (481 smokers; 625 nonsmokers) volunteered information about their gender, age, and political ideology. Among smokers, 52.6% identified their gender as ‘Male’, 46.9% identified as ‘Female’, and 0.004% identified as ‘Non-binary’. A similar distribution was observed among nonsmokers. Among nonsmokers, 50.4% identified as ‘Male’, 48.9% identified as ‘Female’, and 0.006% identified as ‘Non-binary’. We also find similarities in the age distribution across smokers and nonsmokers. Among smokers, 5.9% identified as between 18-24 years old, 42.3% identified as between 25-34 years old, 27% identified as between 35-44 years old, 15.9% identified as between 45-54 years old, 7.2% identified as between 55-64 years old, and 1.2% identified as above 65 years old. Among nonsmokers, 6% identified as between 18-24 years old, 41.5% identified as between 25-34 years old, 26.6% identified as between 35-44 years old, 14% identified as between 45-54 years old, 9.5% identified as between 55-64 years old, and 2% identified as above 65 years old. Regarding the political ideology among smokers, 48.7% identified as ‘Conservative’, 36.6% identified as ‘Liberal’, and 14.2% identified as ‘Other’. A similar distribution was observed among nonsmokers. Among nonsmokers, 45% identified as ‘Conservative’, 44% identified as ‘Liberal’, 10.2% identified as Independent, and the remainder identified their political ideology as ‘Other’. Based on these descriptive statistics, we conclude that there is no evidence that our groups of smokers and nonsmokers were meaningfully differentiated by other demographic traits in addition to their smoking habits.

**Statistical Information.** Each trial of this study is independent from one another, and each condition within each trial is independent from one another. To compare the estimation error of participants across experimental conditions, we first compute the average estimation error for each experimental condition within each trial, at each round. This approach produces 10 independent, group-level measures of accuracy for each experimental condition. To compare the revisions of smokers and nonsmokers separately within the network conditions, we average across the estimates of smokers and nonsmokers within each network separately for each trial, producing two measures of accuracy for each trial, and 20 datapoints in total for each network condition. The trial-level datapoints for smokers and nonsmokers within each network are not independent, and so any comparisons between smokers and nonsmokers within networks are paired by trial. To compare experimental conditions across trials, we use the nonparametric Wilcoxon rank sum test. When comparing smokers and nonsmokers in the same condition in the same trial, we use the Wilcoxon signed-rank test, where pairing by trial adjusts for nonindependence among smokers and nonsmokers in the same networks. All comparisons using the Wilcoxon test are two-tailed to not only test for the hypothesis that social learning increases accuracy, relative to controls, but also for the possibility that it decreases accuracy [1,2].

To compare subjects’ responses in the post-test survey (see “Survey Design”), all survey questions were structured with ordinal categorical response variables. Using ordinal response variables allowed the categorical responses to each question to be assigned a numerical ranking indicating the intensity of response along the dimension specified by the question (e.g. agreement or perceived level of harm). All comparisons of survey responses among subjects within and between conditions apply the Wilcoxon rank sum test to the survey responses, after converting these responses to their ordinal ranking value. We also use regression techniques to test for the effects of subjects’ smoking status on their survey responses, while holding the estimation error of subjects constant cross conditions. To apply these regression techniques to the network conditions, we use clustered standard errors grouped at the trial-level to adjust for nonindependence among subjects in the same network.

**Supplementary Analyses**

**Counterproductive Effects of Anti-smoking Messages on Smokers’ Bias.** A number of studies have reported that repeated exposures to anti-smoking messages can unexpectedly increase bias in smokers’ estimation of tobacco-related health risks [4–7]. To test whether smokers were more likely to increase, rather than decrease, in error in the control condition, we used regression techniques that take advantage of the independence of subjects’ estimates in the control condition. Using the data for all subjects in the condition, we regressed an interaction term between subjects’ initial error and their smoking status on their absolute change in error from Round 1 to Round 3. We used the interaction term to test the theory that, holding initial estimation error constant, smokers will be more likely to increase in their error than nonsmokers as a result of their smoking-related biases. Indeed, we find that smokers were significantly more likely to increase in their estimation error than nonsmokers in the control condition (*n =* 639, *P* < 0.001, β = 0.44, R^2^ = 0.27, CI [0.34, 0.54]).

As an additional test, we regressed the estimates of smokers across all conditions against their change in error, controlling for (1) the distance of subjects’ initial estimates from their neighborhood mean (in the network conditions), and (2) an interaction between smokers’ initial error and their experimental condition. To adjust for non-independence among smokers in the network conditions, we clustered standard errors at the trial-level. We find that compared to their revisions in the network conditions, smokers’ were significantly more likely to increase in error as a result of independent reflection in the control condition (*n =* 638, *P* < 0.001, β = 0.90, R^2^ = 0.67, CI [0.75, 1.06]). These findings are highly consistent with prior studies reporting that exposure to anti-smoking messages can increase, rather than decrease, bias in smokers’ assessments of tobacco-related health risks.


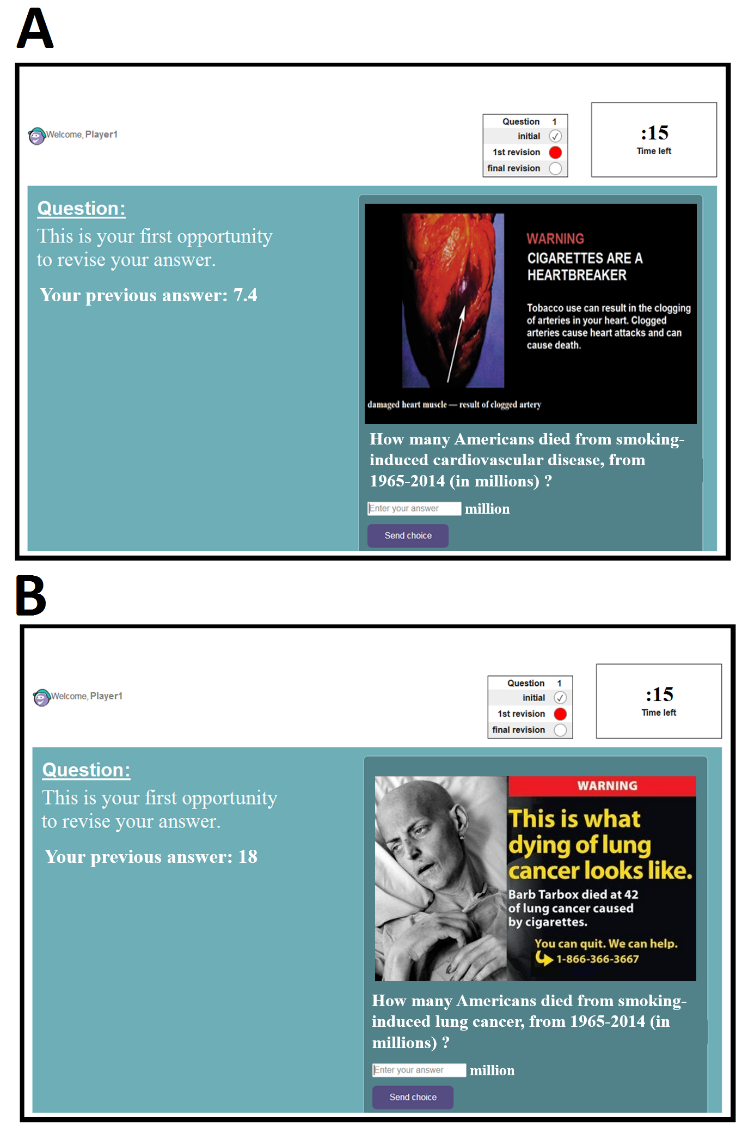


**S2 Fig.** Screenshots of the additional anti-smoking messages used in the robustness trials. (A) An anti-smoking message disseminated by Health Canada as a warning label on cigarette packages, in 2012. The question subjects were asked in response to this advertisement – “How many Americans died from smoking-induced cardiovascular disease, from 1965 – 2014” – was taken from the World Health Organization’s report on the global tobacco epidemic, 2015. (B) A testimony-based anti-smoking message disseminated by Health Canada as a warning label on cigarette packages, in 2012. The question subjects were asked in response to this advertisement – “How many Americans died from smoking-induced lung cancer, from 1965 – 2014” – was taken from the World Health Organization’s report on the global tobacco epidemic, 2015. Permission to display these warning labels has been granted by Health Canada.

**Robustness Trials with Additional Anti-smoking Messages.** To illustrate the robustness of our main results to multiple anti-smoking messages, we replicated our experimental design (Fig 1, main text) using two additional anti-smoking advertisements. Each estimation question was chosen so that, like the question used in our main results (Fig 2, main text), the estimates were expected to be formatted in the millions. For these trials we invited not only new subjects, but also subjects who had already completed the collective intelligence task for the first question in the experiment (Fig 2, main text). Any subject, regardless of whether they completed an earlier question, were randomized across conditions when arriving to the experiment; for this reason, even though subjects were sampled with replacement across questions, the between condition comparisons for each question are internally valid.

In total, we collected 30 experimental groups for Question Two: 6 control smoker groups, 7 control nonsmoker groups, 8 anonymous social networks, and 9 social networks with the smoking status of peers revealed. In response to Question 2 (S2A Fig), subjects were asked to estimate the negative health effects of smoking by answering the question: “How many Americans died from smoking-induced lung cancer, from 1965-2014 (in millions)?” The true answer according to the World Health Organization is 5.4 million.

In total, we also collected 30 experimental groups for Question Three: 6 control smoker groups, 8 control nonsmoker groups, 8 anonymous social networks, and 8 social networks with the smoking status of peers revealed. In response to Question Three (S2B Fig), subjects were asked to estimate the negative health effects of smoking by answering the question: “How many Americans died from smoking-induced cardiovascular disease, from 1965-2014 (in millions)?” The true answer according to the World Health Organization is 7.7 million.


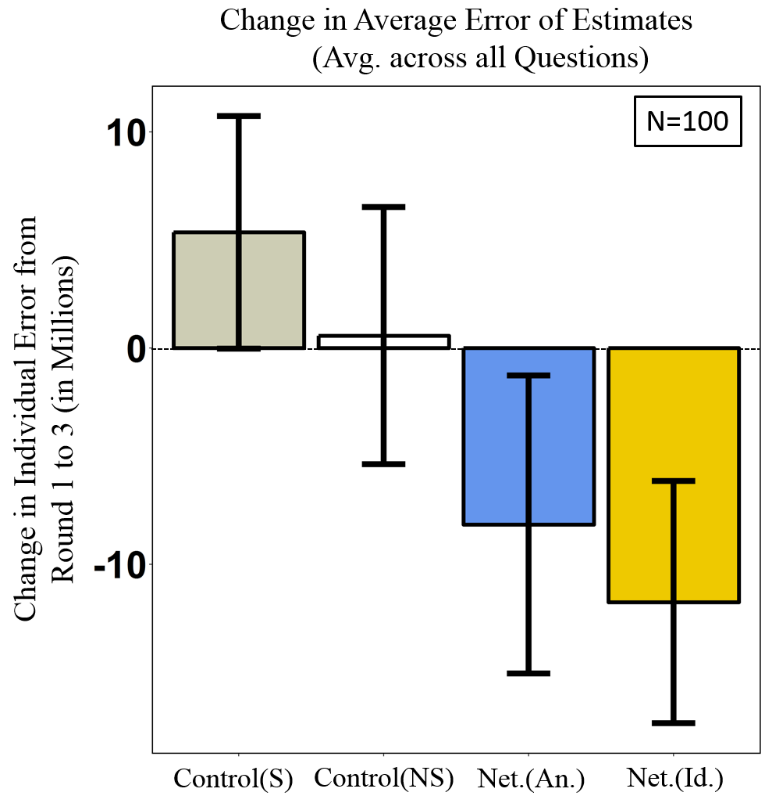


**S3 Fig.** Changes in estimation error across experimental conditions for all questions. Bars display the total change in estimation error from Round 1 to Round 3, averaged across all 10 experimental trials, where each trial provides one observation. All conditions are independent. The error bars show 95% confidence intervals. S, smoker; NS, nonsmoker; An., anonymous; Id., with the smoking status of contacts identified.

In the control condition, there was no significant change in the accuracy of subjects’ estimates of the risks of smoking, for both smokers (*n =* 24; *P* = 0.62, Wilcoxon signed rank test) and nonsmokers (*n =* 23; *P* = 0.11, Wilcoxon signed rank test), suggesting that their baseline inaccuracies were unchanged by individual reflection (S3 Fig). In fact, the average estimation error noticeably increased on average for smokers (by 5.53 million) in the control condition, consistent the counterproductive outcomes of public health campaigns reported in a number of studies. By contrast, in anonymous social networks subjects estimation error was significantly reduced (*n =*26; *P*<0.01, Wilcoxon signed rank test). Similarly, in networks with peers’ smoking status revealed, there was also a significant reduction in subjects’ error when estimating the fatal health effects of smoking (*n*=27; *P*<0.01; Wilcoxon signed rank test).


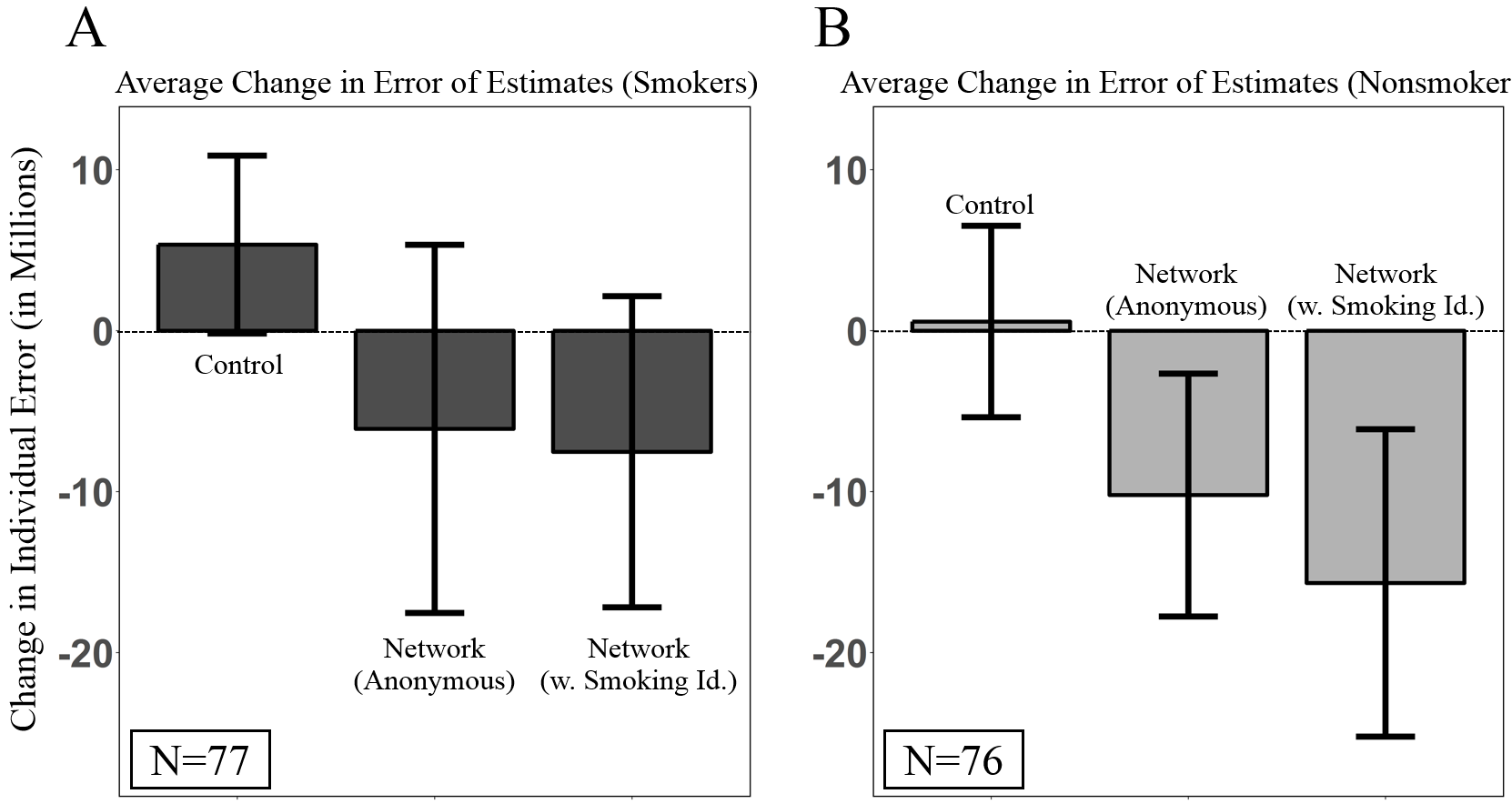


**S4 Fig.** Changes in estimation error across experimental conditions for all questions for both smokers and nonsmokers separately. Bars display the total change in estimation error from Round 1 to Round 3, averaged across all 10 experimental trials, where each trial provides one observation. All conditions are independent. The error bars show 95% confidence intervals.

S4 Fig shows that the benefits of social learning were equally distributed across both smokers and nonsmokers in the network conditions, when averaging across all three questions. Panel A of S4 Fig shows that smokers reduced their error significantly more in both the anonymous networks (*n*=50, *P*<0.05, Wilcoxon rank sum test) and the networks with smoking status revealed (*n*=51, *P*<0.05, Wilcoxon rank sum test), than smokers in the control condition. Similarly, panel B of S4 Fig shows that nonsmokers reduced their error significantly more in both the anonymous networks (*n*=49, *P*=0.01, Wilcoxon rank sum test) and the networks with smoking status revealed (*n*=50, *P*<0.001, Wilcoxon rank sum test), than nonsmokers in the control condition. There was no significant difference in the extent to which smokers and nonsmokers improved in their accuracy across the network conditions (*n*=106, *P*=0.41, Kruskal-Wallis H Test).

**Post-test survey Extended Analysis.** A critique of extant research in the wisdom of the crowds is that changes in subjects’ numeric estimates may not provide direct evidence of changes in their qualitative judgments regarding the estimation problem, particularly in terms of changes in beliefs or attitudes [8,9]. We addressed this issue by inserting a post-test survey after the estimation task, which asked subjects to report their qualitative interpretation of the estimation problem, along with their general beliefs and attitudes toward the negative health effects of tobacco use. Every subject, regardless of condition, was invited to complete the post-test survey immediately after the game. Before the game, subjects were not given any indication that they would be asked to complete a survey at the end of the experiment. For this reason, subjects’ responses to the survey provide an experimentally manipulated outcome measure, where we can test the effects of social learning on subjects’ attitudes and beliefs by comparing the survey responses of subjects in the network conditions to subjects in the control condition. This method allows us to determine, first, the extent to which subjects’ numeric estimates are consistent with their qualitative beliefs and attitudes, and second, whether social learning has the capacity to influence the self-reported beliefs and attitudes of smokers and nonsmokers. Because our robustness trials involved repeat subjects who participated in multiple estimation tasks, we only asked subjects to complete the survey after the first anti-smoking message (Fig 2, main text).

The post-test survey consisted of seven questions, which were always presented in the same order to every subject, regardless of condition (see “Survey Design”). For statistical comparisons, the possible responses to each question were formatted to an ordinal scale, so that the categorical inputs could be converted to numerically ranked values that could be compared using the nonparametric Wilcoxon test, and so that they could be aggregated at the trial-level to produce independent data points.

For Question 1, immediately after the estimation task, subjects were asked to rank their confidence in the accuracy of their estimates using a 7-point Likert-scale in response to the following question: “During the game, how confident were you in your estimates of how many people will die of tobacco use in developed countries, in 2030?” (see “Survey Design”).

Questions 2 and 3 assessed subjects’ general beliefs regarding the negative health risks associated with tobacco use. For Question 2, subjects answered the question, “Do you agree that smoking tobacco has negative and potentially fatal consequences for personal health?”, using a standard 5-point scale from “strongly disagree” to “strongly agree”. For Question 3, subjects answered the question “Do you agree that tobacco use poses serious risks for society?” using the same 5-point Likert scale indicating agreement.

To detect intergroup bias (and the potential effects of social learning on intergroup bias), we asked subjects a number of questions to reveal their attitudes toward people with the opposite smoking status to their own. Using a 5-point Likert scale to indicate agreement in each case (“survey design”), subjects were asked the following questions in order: Question 4, “Do you agree that smokers and nonsmokers conflict in their views about the health effects of smoking?”; Question 5, “Do you agree that smokers are more likely to misinterpret data about the health effects of smoking?”; and Question 6, “Do you agree that nonsmokers are more likely to misinterpret data about the health effects of smoking?” (“Survey Design”).

As an additional measure of intergroup bias of particular relevance to social learning, we also gauged subjects’ willingness to communicate about tobacco use with people with opposite smoking habits to their own. For this purpose, we adapted a standard survey question from the literature on political polarization [10], where subjects are asked to describe their preferred social group composition for future discussions on political topics. In Question 7, we asked subjects the following question – “In the future, if you were to discuss the health effects of tobacco use in a group with other people, what kind of group would you be most interested in joining? A group with:” – to which subjects answered using a 7-point Likert-scale, from “all smokers” to “all nonsmokers”, with a neutral middle point of “equal numbers of smokers and nonsmokers” (“Survey Design”).

A foundational assumption in the wisdom of crowds literature is that social learning is driven by the phenomenon where more accurate individuals revise their answers less as a result of their presumed confidence [1, 11, 12]. However, despite no significant differences in the initial and final accuracy of smokers and nonsmokers in the control condition (*n* = 20; *P* = 0.19, Wilcoxon rank sum test), anonymous networks (*n* = 20; *P* = 0.31, Wilcoxon rank sum test), and networks with smoking status revealed (*n* = 20; *P* = 0.63, Wilcoxon rank test), smokers reported being more confident in their accuracy across all conditions: in the control condition (*n* = 20; P < 0.01, Wilcoxon signed rank), the anonymous network condition (*n* = 20; P = 0.01, Wilcoxon signed rank), and the networks with smoking status revealed (*n* = 20; P<0.01, Wilcoxon signed rank).

To test for robustness, we regressed subjects’ initial and final estimation error on their self-ascribed confidence, while clustering standard errors at the trial-level to adjust for nonindependence among subjects in the same networks. With this approach, we continue to observe that smokers reported significantly higher confidence levels than nonsmokers in the control condition (*n =* 532, *p*<0.001, CI [0.19, 0.75]), the anonymous networks (*n =* 334, *p*<0.001, CI [0.34, 1]), and the networks with subjects’ smoking status revealed (*n =* 328, *p*<0.001, CI [0.43, 1.13]). This finding raises an important question for wisdom of the crowd literature, as it suggests that numeric accuracy cannot be taken as a reliable signal of subjects’ perceived confidence in their estimate, especially when biased reasoning is at play [13–18].

When asked to evaluate the general severity of health risks posed by tobacco use in Question 2, smokers’ ratings were significantly lower than nonsmokers’ in the control condition (*n* = 20; *P* < 0.01, Wilcoxon rank sum test), the anonymous network condition (*n* = 20; *P* = 0.01, Wilcoxon rank sum test), and the networks with smoking status revealed (*n* = 20; *P* < 0.01, Wilcoxon rank sum test). Similarly, when asked to evaluate the health risks that tobacco use poses for society in Question 3, smokers’ ratings were also significantly lower than nonsmokers’ in the control condition (*n* = 20; *P* < 0.01, Wilcoxon rank sum test), the anonymous network condition (*n* = 20; *P* < 0.01, Wilcoxon rank sum test), and the networks with smoking status revealed (*n* = 20; *P* < 0.01, Wilcoxon rank sum test).

In response to Question 4, we find that social learning in the networks with smoking status revealed further reduced subjects’ biased judgements toward the accuracy of smokers. Nonsmokers’ responses to Question 4 were significantly higher than smokers’ responses in the control condition (*n* = 20; *P* = 0.01, Wilcoxon rank sum test) and the anonymous networks (*n* = 20, *P* = 0.03, Wilcoxon rank sum test), indicating that in these networks, nonsmokers retained a significant bias toward smokers. However, after interacting in the social networks with the smoking status of peers revealed, there was no significant difference in smokers’ and nonsmokers’ perceptions of smokers’ capacity to interpret health-related scientific information (*n* = 20, *P* = 0.19, Wilcoxon rank sum test). These results suggest that revealing the smoking status of peers in the network conditions significantly reduced intergroup bias between nonsmokers and smokers as concerns their views about one another’s capacity to accurately interpret tobacco-related scientific information.

In response to Question 5, smokers’ reported preferring social groups consisting of equal numbers of smokers and nonsmokers when discussing tobacco-related health information (mean response in the control condition, 4.04; in the anonymous networks, 4.19; and in the networks with smoking status revealed, 3.99). Nonsmokers, on the other hand, were more likely to prefer social groups with slightly more nonsmokers (mean response in the control condition, 3.38; in the anonymous networks, 3.29; and in the networks with smoking status revealed, 3.36). The preferences of smokers and nonsmokers as reported for Question 7 were significantly different in each condition: the control condition (*n* = 20, *P* < 0.01, Wilcoxon rank sum test), the anonymous network (*n* = 20, *P* < 0.01, Wilcoxon rank sum test), the network with subjects’ smoking status revealed (*n* = 20, *P* < 0.01, Wilcoxon rank sum test). In general, however, smokers’ and nonsmokers’ preferences overlapped strongly on the preference for social groups consisting equally of smokers and nonsmokers. This similarity in preference for balanced social groups is consistent with the finding that revealing smoking status during networked interactions can enhance social learning and the reduction of intergroup bias.

**Survey Design.** This survey was designed using standard measures of health-related attitudes [19, 20]. We introduced several novel measures to adapt health-related surveys to the framework of estimation tasks. To assess subjects’ confidence in their estimates, we employed a 7 point Likert scale that subjects used to rank their confidence. To assess intergroup bias among smokers and nonsmokers, we asked subjects to express their preferred social group composition for future discussions of the negative health effects of smoking (see Question 5). This question was adapted from the literature on political polarization [10], where it has been used to robustly measure intergroup bias in terms of the willingness of subjects to exchange information on political topics with members of an opposing partisanship. All survey questions were presented in horizontal orientation from left-to-right, where the answer with the lowest ranking appeared on the leftmost end of the horizontal scale. The survey was constructed using Qualtrics. After subjects finished the estimation task, they arrived at a final page that displayed their financial earnings along with an embedded link to the survey. Subjects were told that the survey was voluntary.

**Survey Questions**

1. During the game, how confident were you in your estimates of how many people will die of tobacco use in developed countries, in 2030?

o Not confident at all

o Unconfident

o Somewhat unconfident

o Neither confident or unconfident

o Somewhat confident

o Confident

o Absolutely Confident

2. Do you agree that smoking tobacco has negative and potentially fatal consequences for personal health?

o Strongly disagree

o Disagree

o Neutral

o Agree

o Strongly agree

3. Do you agree that tobacco use poses serious health risks for society?

o Strongly disagree

o Disagree

o Neutral

o Agree

o Strongly agree

4. Do you agree that smokers are more likely to misinterpret data about the health effects of smoking?

o Strongly disagree

o Disagree

o Neutral

o Agree

o Strongly agree

5. In the future, if you were to discuss the health effects of tobacco use in a group with other people, what kind of group would you be most interested in joining? A group with:

o All nonsmokers

o Mostly nonsmokers

o Slightly more nonsmokers

o Equal smokers & nonsmokers

o Slightly more smokers

o Mostly smokers

o All smokers

**Supplementary References**

1. Becker J, Brackbill D, Centola D. Network dynamics of social influence in the wisdom of crowds. PNAS. 2017;114: 5070–5076.

2. Guilbeault D, Becker J, Centola D. Social learning and partisan bias in the interpretation of climate trends. PNAS. 2018;115: 9714–9719.

3. Orentlicher D. The FDA’s Graphic Tobacco Warnings and the First Amendment. New England Journal of Medicine. 2013;369: 204–206.

4. Henriksen L, Dauphine A, Wang Y, Fortmann S. Industry sponsored anti-smoking ads and adolescent reactance: test of a boomerang effect. Tobacco Control. 2006;15: 13–18.

5. McVey D, Stapleton J. Can anti-smoking television advertising affect smoking behaviour? Controlled trial of the Health Education Authority for England’s anti-smoking TV campaign. Tobacco Control. 2000;9: 273–282.

6. Niederdeppe J, Fiore M, Baker T, Smith S. Smoking-Cessation Media Campaigns and Their Effectiveness Among Socioeconomically Advantaged and Disadvantaged Populations. Am J Public Health. 2008;98: 916–924.

7. Gibson L, Parvanta S, Jeong M, Hornik R. Evaluation of a mass media campaign promoting using help to quit smoking. Am J Prev Med. 2014;46: 487–495.

8. Kao B, Berdahl A, Hartnett A, Lutz M, Bak-Coleman J, Ionannou C, et al. Counteracting estimation bias and social influence to improve the wisdom of crowds. J Roy Soc Int. 2018; 15.

9. Pedersen R, Mutz D. Attitudes Toward Economic Inequality: The Illusory Agreement. Pol Sci Res Met. 2018: 1–17

10. Levendusky M. Americans, Not Partisans: Can Priming American National Identity Reduce Affective Polarization? J of Pol. 2017;80: 59–70.

11. March J, Simon H. Organizations. New York: Wiley-Blackwell; 1993.

12. Prelec D, Seung H, McCoy J. A solution to the single-question crowd wisdom problem. Nature. 2017;541: 532–535.

13. Bénabou R, Tirole J. Self-Confidence and Personal Motivation. Q J Econ. 2002;117: 871–915.

14. Horry R, Colton L, Williamson P. Confidence–accuracy resolution in the misinformation paradigm is influenced by the availability of source cues. Acta Psychologica. 2014;151: 164–173.

15. Lebreton M, Abitbol R, Daunizeau J, Pessiglione M. Automatic integration of confidence in the brain valuation signal. Nat Neuros. 2015;18: 1159–1167.

16. Masiero M, Lucchiari C, Pravettoni G. Personal Fable: Optimistic Bias in Cigarette Smokers. Int J High Risk Behav Addict. 2015;4: e20939.

17. Masiero M, Riva S, Oliveri S, Fioretti C, Pravettoni G. Optimistic bias in young adults for cancer, cardiovascular and respiratory diseases: A pilot study on smokers and drinkers. J Health Psychol 2006;23: 645–656.

18. Segerstrom S, Mcarthy W, Caskey N, Gross T, Jarvik M. Optimistic Bias Among Cigarette Smokers. J App Soc Psych. 1993;23: 1606–1618.

19. Hornik R. Public Health Communication. New York: Routledge; 2002.

20. Strasser A, Cappella J, Jepson C, Fishbein M, Tang K, Han E, Lerman C. Experimental evaluation of antitobacco PSAs: Effects of message content and format on physiological and behavioral outcomes. Nicotine Tob Res. 2009;11: 293–302.
