# Supplementary figures and images for: Networked collective intelligence improves dissemination of scientific information regarding smoking risks
Source: PLoS One. 2020 Feb 6;15(2):e0227813. doi: 10.1371/journal.pone.0227813 (PMC7004329; doi:10.1371/journal.pone.0227813)

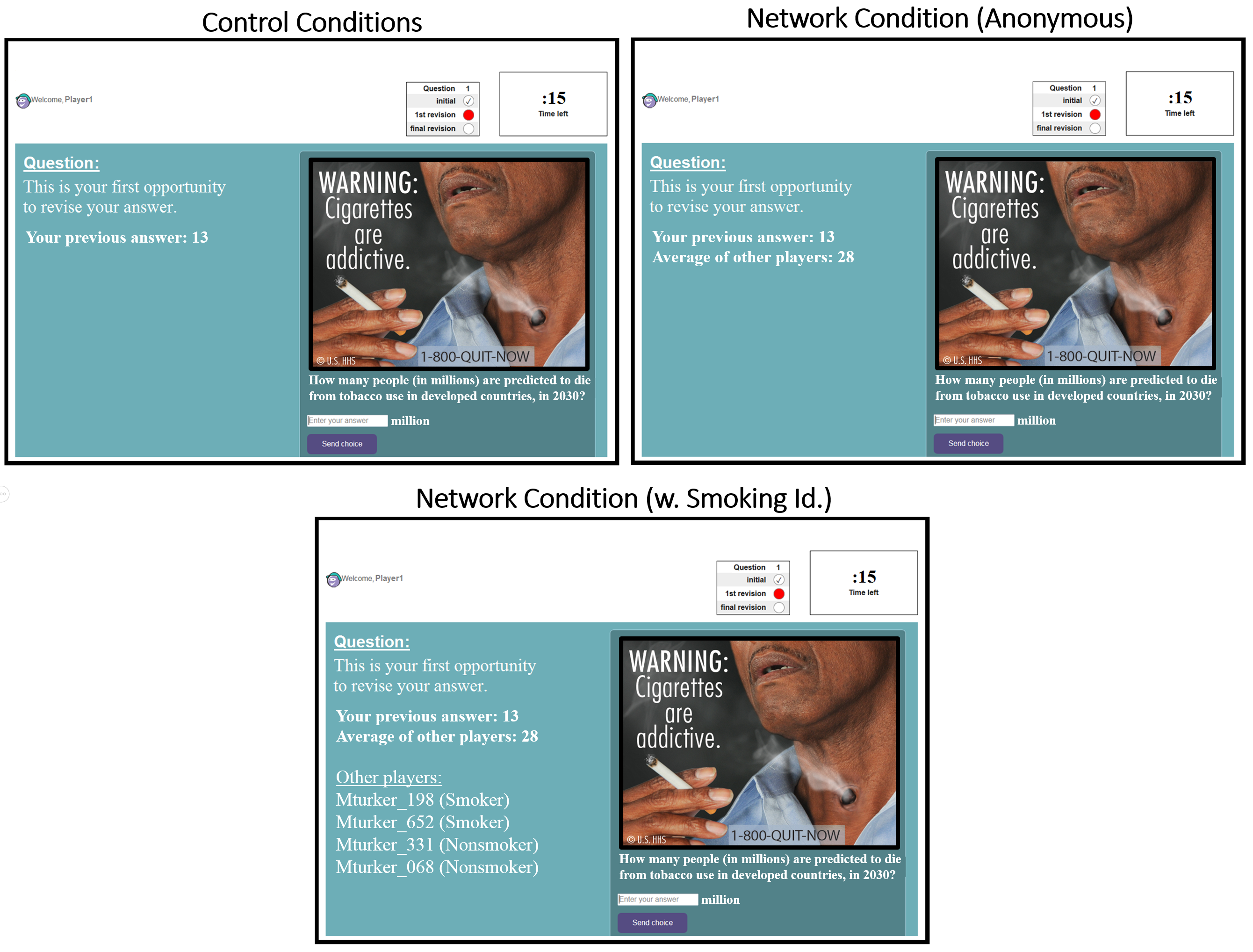

Supplement: S1 Fig — (TIFF) [file pone.0227813.s002.tiff]

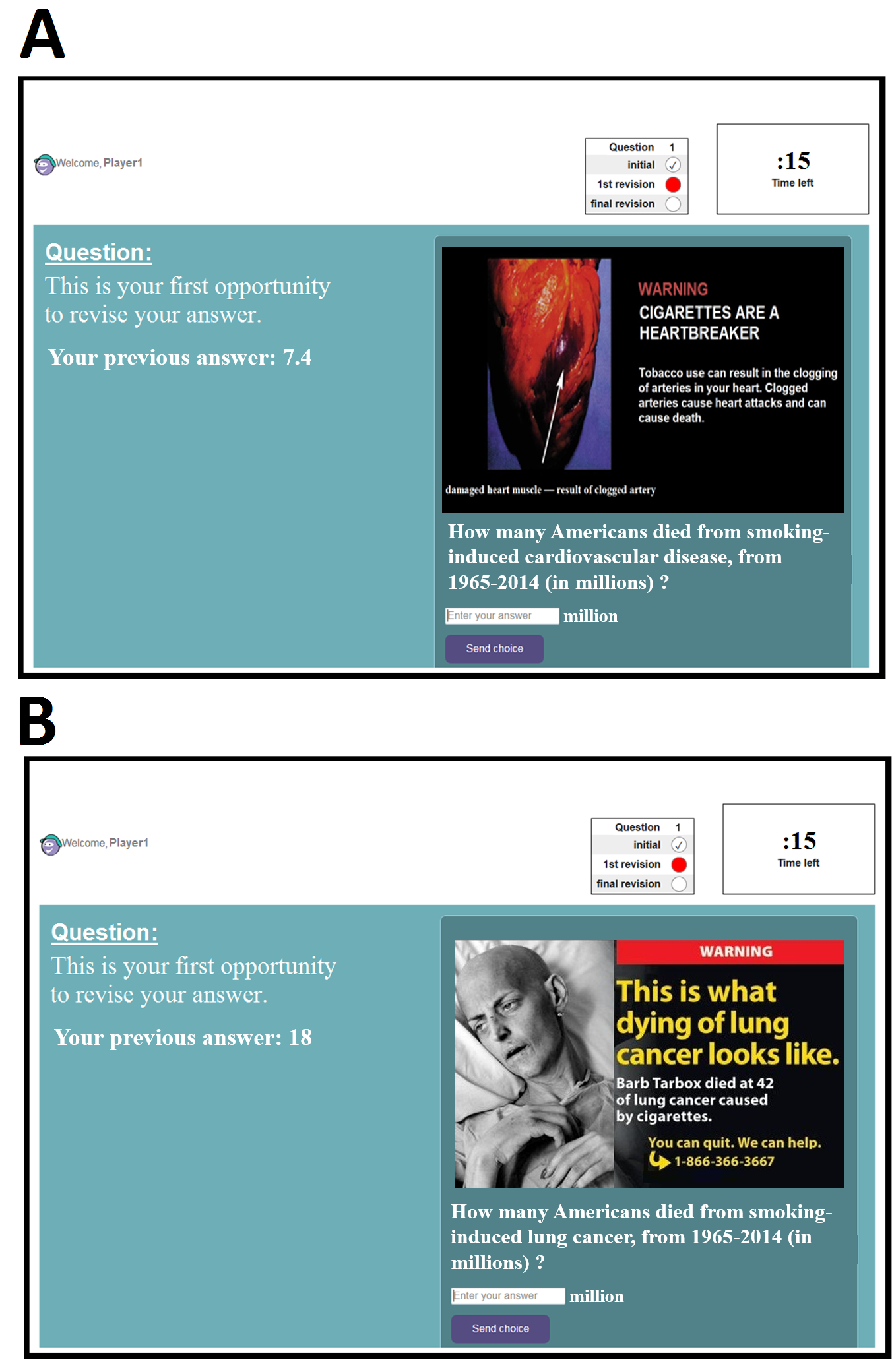

Supplement: S2 Fig — (TIFF) [file pone.0227813.s003.tiff]

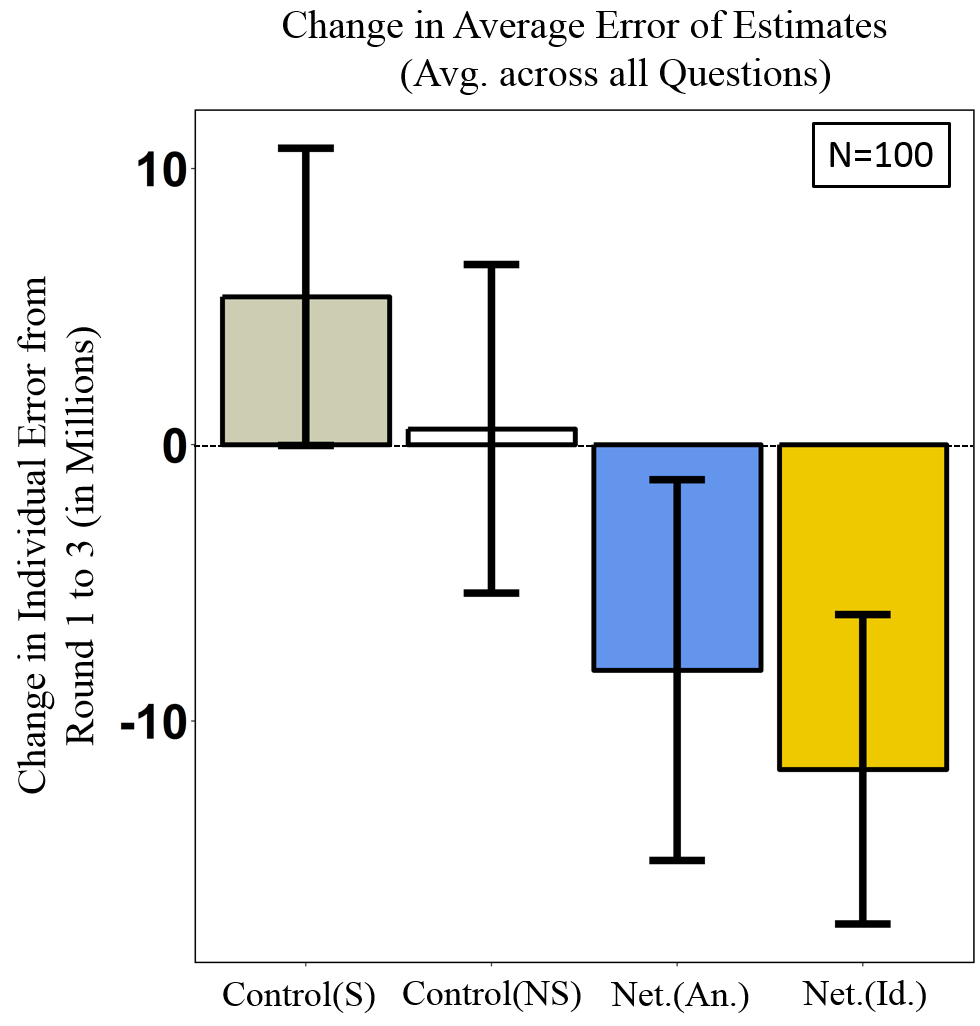

Supplement: S3 Fig — (TIFF) [file pone.0227813.s004.tiff]

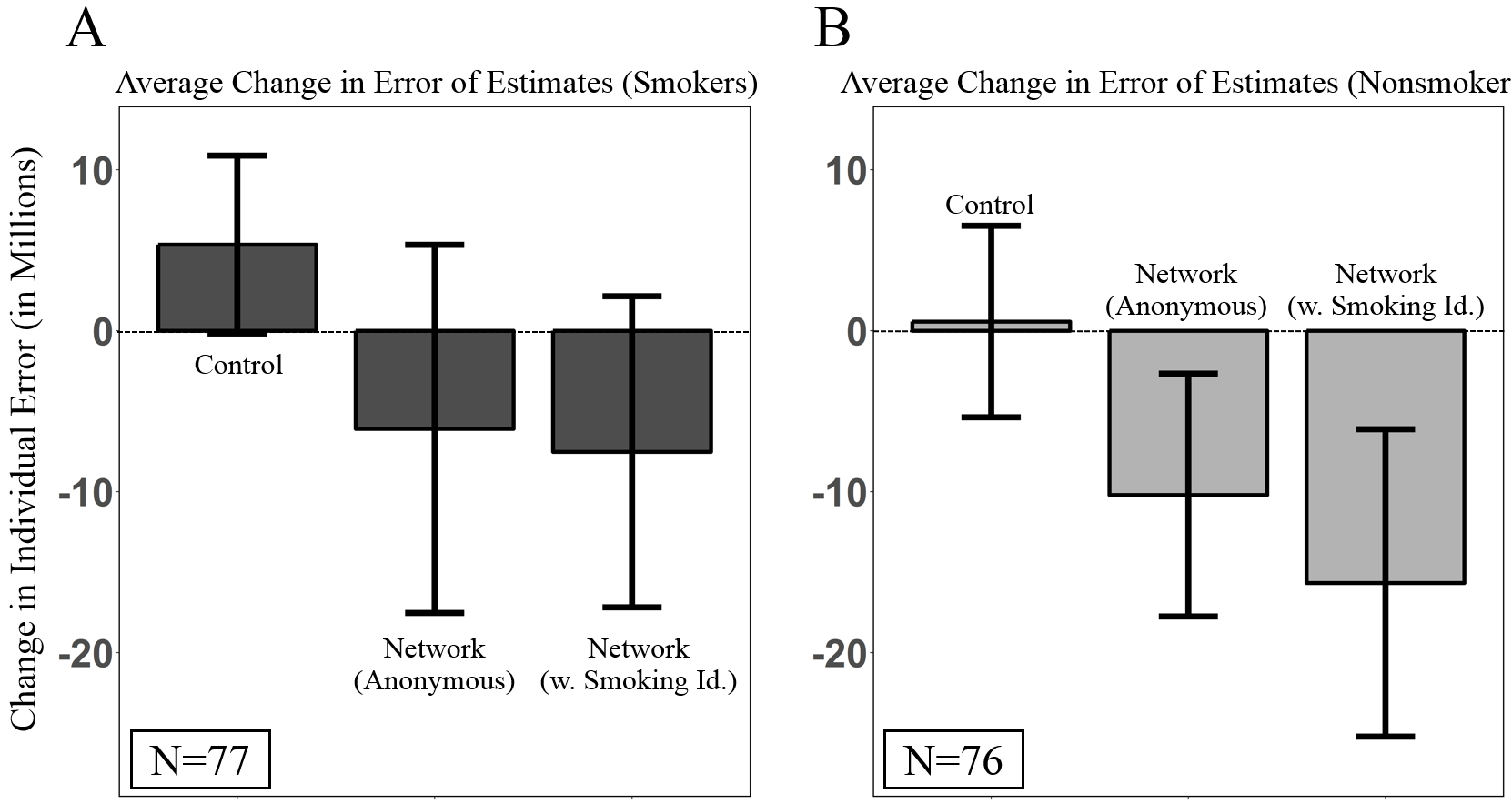

Supplement: S4 Fig — (TIFF) [file pone.0227813.s005.tiff]
